# Online class-related hope and learning engagement among college students: the mediating role of external motivation and the moderating role of gender

**DOI:** 10.3389/fpsyg.2026.1856483

**Published:** 2026-08-28

**Authors:** Youlai Zeng, Xuemin Zhao, Nan Sun

**Affiliations:** Faculty of Education, Liaoning Normal University, Dalian, China

**Keywords:** control-value theory of achievement emotions, external motivation, learning engagement, online class-related hope, self-determination theory

## Abstract

**Introduction:**

The widespread adoption of online classes has drawn attention to factors associated with students’ learning engagement, with the role of hope becoming increasingly salient. Although hope is recognized as important in education, the mechanism through which online class-related hope relates to learning engagement remains underexplored.

**Methods:**

This cross-sectional study examined the associations among online class-related hope, external motivation, and learning engagement, and further tested the mediating role of external motivation and the moderating role of gender. A total of 1,294 college students from multiple universities across China completed self-report questionnaires measuring online class-related hope, motivation, and learning engagement. Grounded in the Control-Value Theory of Achievement Emotions and Self-Determination Theory, we tested a moderated mediation model via the SPSS PROCESS macro (Models 4 and 14).

**Results:**

The results revealed a significant positive association between online class-related hope and learning engagement. External motivation partially mediated this relationship, whereas gender did not significantly moderate the path from external motivation to learning engagement.

**Discussion:**

These findings provide preliminary evidence that hope and external motivation jointly predict learning engagement in online settings, extending the two theoretical frameworks to online learning contexts. The absence of meaningful gender moderation offers new complementary evidence amid inconsistent prior research outcomes. Given the cross-sectional design, causal inferences cannot be drawn, and relevant instructional recommendations await further longitudinal verification. This study clarifies the conditional process linking online class-related hope to learning engagement and lays a nuanced foundation for subsequent research.

## 1 Introduction

The rapid advancement of digital technology has propelled online classes into a normalized component of modern higher education. Online classes feature high autonomy, situational openness, and reduced in-person interaction, making students’ learning engagement susceptible to monotonous course content and insufficient offline communication, thereby highlighting the necessity of exploring the underlying psychological mechanisms that sustain students’ online leaning engagement. Scholars have increasingly focused on emotional and motivational antecedents of college students’ learning engagement, as affective and motivational factors are core psychological resources that determine academic persistence and behavioral investment. Existing evidence demonstrates that adaptive emotional functioning during learning is a prerequisite for successful learning (Amriza et al., 2024). As a positive achievement emotion, hope exerts a beneficial influence on students’ learning behaviors and academic development (Ge et al., 2025; Zeinalipour, 2022; Wong and Cheung, 2024). Hope refers to a positive psychological resource that enables individuals to formulate goal-directed determination and actionable pathways to pursue academic targets (Snyder, 2002; Snyder et al., 1991). Students with higher hopeful thinking are more capable of generating flexible coping strategies, overcoming academic obstacles, and maintaining continuous learning investment (Irving et al., 2004; Snyder et al., 2016; Risse et al., 2018), which further promotes adaptive academic performance and psychological well-being (Pleeging et al., 2021).

Learning motivation is also a robust predictor of learning engagement across diverse educational environments (Burnette et al., 2013; Gottfried et al., 2013; Huang, 2011; Seaton et al., 2014). Student-centered online classes require sustained voluntary participation and self-regulated learning, which makes motivational regulation particularly critical for learning quality (Hartley and Bendixen, 2001). Rather than generalizing that online classes universally weakens intrinsic motivation, motivational patterns vary considerably across online classes and individual learners. In many self-directed online scenarios where situational interest is limited, students may exhibit relatively lower intrinsic learning inclination, allowing external motivation forces to play a more prominent role in driving goal-oriented academic behaviors (Layaman et al., 2021; Lin et al., 2019). External motivation bridges individual psychological traits and observable learning behaviors, effectively facilitating structured and persistent academic engagement when internal interest is insufficient. Prior literature has validated positive correlations between general trait hope and offline learning engagement (Ge et al., 2025; Marques et al., 2017; Tomás et al., 2020), yet few studies distinguish context-specific hopeful emotions unique to online classes.

This study defines online class-related hope as a situational achievement emotion rooted in Pekrun’s Control-Value Theory, emerging from learners’ dual cognitive evaluations of online classes: perceived control over personal learning progress and perceived value of course content (Pekrun, 2006; Pekrun et al., 2017). When students judge online classes controllable and valuable, they generate anticipatory positive emotion featuring goal agency and pathway planning—namely online class-related hope. Unlike stable trait hope measured in offline settings, this construct is state-specific and tightly bound to online classes stimuli, filling the gap of context-specific emotional research within Control-Value Theory. In this study, the online learning context consists of synchronous live streaming lectures and asynchronous self-learning resources without frequent face-to-face communication, characterized by delayed teacher-student interaction, sparse peer feedback, and separated physical space between learners and instructors. Control-Value Theory explains how achievement emotions arise from control-value appraisals and regulate subsequent academic cognition and behavior, while Self-Determination Theory systematically classifies motivational regulations and their behavioral consequences (Ryan and Deci, 2000). The two theoretical frameworks form a coherent logical chain for the current conditional process model: online class-related hope (a positive achievement emotion derived from control-value evaluation) first shapes learners’ motivational regulation strategies, specially boosting external motivation that compensates insufficient intrinsic interest in low-interaction online classes; external motivation thereafter translates emotional anticipations into tangible learning behavioral investment, namely learning engagement. Although intrinsic motivation occupies a core theoretical position in Self-Determination Theory, online classes’ weak interpersonal feedback often fails to activate spontaneous internal interest. External motivation, especially identified regulation where students recognize the instrumental value of online courses, delivers unique explanatory power for sustained students’ learning engagement, justifying its selection as the mediating variable instead of intrinsic motivation or other motivational subtypes.

This integrated theoretical logic avoids disjointed separate introductions of two theories and builds tight logical links across theoretical frameworks, core emotional variable, motivational mediator, and outcome variable. Defined as sustained physical, cognitive, and emotional energy devoted to academic activities (Oh et al., 2017). Learning engagement directly predicts academic achievement and reduces dropout risks among online learners (Brozina et al., 2019; Hughes et al., 2020; Wang et al., 2019), making it a vital outcome indicator for online educational psychology research.

Gender is incorporated as a moderator based on consistent gender disparities in emotional sensitivity and motivational response documented in prior educational psychology work. Female students demonstrate stronger emotional perception, more proactive academic self-regulation, and higher responsiveness to external motivational incentives; in contrast, male students’ learning behaviors rely more heavily on spontaneous intrinsic interest (Freudenthaler et al., 2008; Heylen et al., 2012; Ong and Lai, 2006; Voyer and Voyer, 2014). Such stable individual differences generate divergent strength in the external motivation-learning engagement association: external motivation predicts engagement more strongly for female learners, while the association is weaker among male learners.

This study delivers sharp, targeted incremental contributions to educational psychology within online learning contexts by connecting achievement emotion theory, motivation theory, online class-related hope, external motivation, gender disparities, and learning engagement. First, this study operationalizes online class-related hope as a situational achievement emotion unique to online learning settings, differentiating it from dispositional trait hope and broadening the applicable scope of Control-Value Theory. Second, this study integrates Control-Value Theory and Self-Determination Theory into an integrated emotion-motivation pathway framework. It establishes clear logical links between core theoretical viewpoints and research variables, thereby resolving the fragmented theoretical analyses found in existing literature. Third, this study elaborates on the theoretical basis for treating identified regulation (a subtype of external motivation) as the mediator, thereby addressing the intrinsic-motivation bias prevalent in existing research on online class motivation. Fourth, this study constructs a full theoretical framework of gender moderation, explaining how gender shapes the link between external motivation and learning engagement, and uncovering the boundary conditions of the proposed emotion-motivation model.

Collectively, this research unpacks the mediated and moderated conditional process linking online class-related hope to college students’ learning engagement, providing systematic theoretical extensions and rigorous empirical evidence for online education psychology. Based on the integrated theoretical reasoning above and identified research gaps, three core research objectives are proposed: to test the direct correlation between online class-related hope and learning engagement; to verify the serial indirect pathway where online class-related hope predicts external motivation, and external motivation further predicts learning engagement; to examine whether gender moderates the pathway from external motivation to learning engagement. To refine hypothesis development and separate combined predictive and mediating relationships into clear sub-hypotheses, this study puts forward four distinct hypotheses as follows:

*Hypothesis 1*: College students’ online class-related hope is positively associated with their learning engagement.

*Hypothesis 2a*: Online class-related hope positively predicts college students’ external motivation.

*Hypothesis 2b*: External motivation plays a mediating role in the positive relationship between online class-related hope and learning engagement.

*Hypothesis 3*: Gender moderates the positive association between external motivation and learning engagement; this correlation is significantly stronger for female students than male students.

**Figure 1:**
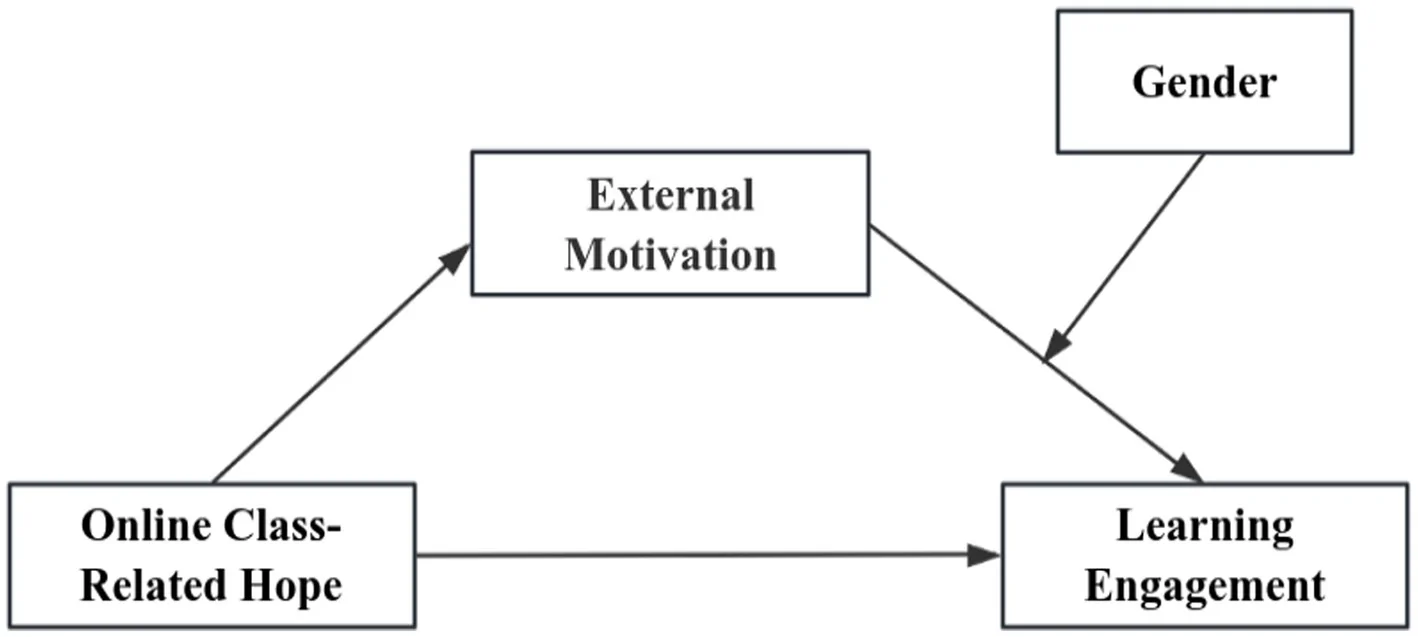
The hypothesized moderated mediation model.

In summary, the theoretical model with moderated mediating effects constructed in this study is shown in Figure 1.

## 2 Materials and methods

### 2.1 Participants and survey procedures

This study adopted a stratified convenience sampling design. To capture the diverse online learning contexts of Chinese undergraduates, we recruited participants from multiple universities across Northeast, East, and Central China. Three core sampling dimensions were used to ensure diverse participant backgrounds: (1) geographic coverage spanning three major regions of China; (2) disciplinary coverage encompassing the humanities, natural sciences, engineering, medicine, economics, management, and teacher education; (3) institutional type coverage including comprehensive research universities and industry-specialized universities.

This research obtained institutional ethical approval before data collection began. Questionnaire recruitment was carried out with support from course instructors and student counselors. An anonymous online questionnaire hosted on Wenjuanxing was distributed through class WeChat groups and campus online forums. Participation was completely voluntary; no mandatory in-class completion was required, and participants could withdraw from the survey at any time without negative repercussions. All respondents completed the questionnaire independently on their personal mobile devices after class. Group in-class administration was avoided to reduce social desirability bias stemming from administrative pressure. No monetary rewards were provided to participants.

In total, 1,400 students opened the questionnaire link. Raw data were screened rigorously based on predefined standardized inclusion and exclusion criteria. Inclusion criteria were as follows: (1) full-time undergraduate students; (2) age 18 years or older; (3) provision of online informed consent before filling out the questionnaire; (4) formal online course learning experience in the prior academic year. Exclusion criteria included careless response patterns such as straight-lining and mechanical repetitive answering, response durations shorter than the 3-min minimum valid completion threshold confirmed in the pilot study, and incorrect responses to two embedded attention-check items. Following these screening standards, 106 invalid and unqualified questionnaires were removed, yielding 1,294 valid responses and an effective response rate of 92.4%. The final sample achieved multidimensional coverage of undergraduate populations and sufficiently reflected regional and disciplinary heterogeneity. Detailed sample distribution is shown in Table 1.

**Table 1:** Demographic characteristics of participants.

Sample dimension	Category	Sample size (n)
Disciplinary grouping	Humanities and Social Sciences	658
	Engineering and Medical Sciences	636
Academic year	Freshman (Year 1)	394
	Sophomore (Year 2)	445
	Junior (Year 3)	101
	Senior (Year 4)	354
Academic performance	Top 20%	177
	21–40%	325
	41–60%	500
	61–80%	214
	Bottom 20%	78
Gender composition	Male	368
	Female	926

In addition, the sample exhibited an unbalanced gender distribution. To disentangle its potential confounding effects on moderation analyses, systematic examinations were conducted based on real-world demographic patterns and multiple statistical robustness tests. Demographically, the overrepresentation of female participants aligns with the actual gender enrollment structure for humanities, social sciences, and teacher education majors at Chinese universities, which formed the primary study population. This gender composition reflects inherent demographic characteristics of the target population and does not introduce systematic gender-related sampling bias. Furthermore, the sample achieved balanced coverage across geographic regions, disciplines, academic years, and academic performance. Overall sample diversity remained satisfactory, with imbalance limited only to gender. Multiple statistical results further confirmed that the disparity in male and female subgroup sizes would not compromise the validity and stability of moderation findings.

First, the SPSS PROCESS macro was used to run bootstrap-based regression moderation analysis, an approach statistically robust to unequal group sizes. A total of 5,000 bias-corrected bootstrap resamples were employed to ensure accurate confidence interval estimation under unbalanced grouping conditions. Second, two complementary robustness strategies were implemented. We randomly matched female participants to create a gender-balanced subsample (*N* = 736), and we also ran weighted regression on the full sample (*N* = 1,294) to equalize the statistical weight of male and female respondents without removing any observations. The identical Model 14 moderated mediation model was re-estimated for both supplementary datasets. All three datasets—the original unweighted full sample, the matched balanced subsample, and the weighted full sample—yielded highly consistent non-significant interaction results. In the weighted full sample, the interaction term between external motivation and gender was *B* = 0.040, *p* = 0.238, 95% CI [−0.027, 0.107]. The index of moderated mediation also included zero across all three analyses. These parallel outcomes strongly indicate that gender imbalance did not distort the core moderating pathway estimates.

In summary, although the gender ratio was uneven, the sample comprehensively captured undergraduates from Northeast, East, and Central China with diverse geographic backgrounds, disciplines, academic years, and levels of academic performance. The gender disparity exerted no harmful influence on the reliability and generalizability of results yielded by the conditional process model.

### 2.2 Ethical approval

This study was conducted in full compliance with ethical standards for human subjects research and received ethical approval from the Ethics Committee of Liaoning Normal University (Approval No. LL2026180). The committee verified that this research satisfied all relevant ethical criteria. All participants took part in the survey entirely on a voluntary basis. All collected data were de-identified to protect participants’ personal confidentiality.

### 2.3 Measures

The core variables of this study include online class-related hope, external motivation, and learning engagement. All measurement tools were developed following standardized workflows: translation and revision, preliminary item screening, and comprehensive psychometric validation. Psychometric analyses were conducted using a sample of 1,294 valid survey responses, and common method bias was effectively controlled. Reliability and validity results confirmed that all scales produce high-quality data suitable for subsequent structural modeling and hypothesis testing. The detailed test results of each variable are presented as follows.

#### 2.3.1 Online Class-Related Hope Scale

This study adopted the hope subscale of the Achievement Emotions Questionnaire (AEQ) (Pekrun et al., 2002) as its primary measurement tool. Grounded in control-value theory, this widely used multidimensional self-report scale for academic emotion research covers three core learning scenarios (in-class learning, self-regulated learning, and examinations) and assesses eight distinct academic emotions including enjoyment, hope, anxiety, and hopelessness, enabling stable measurement of students’ academic emotional traits. Supported by standardized administration procedures and abundant empirical reliability and validity evidence from Pekrun et al. (2002), the instrument was deemed appropriate for capturing pre-class online class-related hope in the current research, so we revised the original scale through a standardized three-step translation, back-translation, and expert review process to align with online teaching contexts. Two researchers with psychology and English backgrounds independently completed initial translations to guarantee semantic accuracy and fit formal Chinese academic expression, after which a senior English linguistics professor carried out back-translation; iterative comparison and discussion of ambiguous wording between the original and back-translated versions fully preserved the core meaning of every original item. Five university instructors with extensive online teaching experience then reviewed the drafted Chinese scale, refining item wording for readability and compatibility with online learning environments and removing items inconsistent with the research setting to form an initial draft of the adapted scale.

The core revision work centered on rewriting offline classroom descriptions to match online learning scenarios, such as adjusting the original item “I am confident that I will understand the material in this class” to “I am confident that I can grasp the content taught in online classes.” Psychometric guidelines note that single items cannot form stable latent variable dimensions, and preliminary screening revealed that the original hope items corresponding to in-class, after-class, and exam contexts all operated as single-item indicators, failing to establish steady dimensions and misaligning with this study’s focus on pre-class online class-related hope, which led to the elimination of these items; seven items specifically measuring pre-class hope were retained to form the final unidimensional Online Class-related Hope Scale. We further invited those five online teaching instructors to conduct expert content validation via a 4-point relevance rating scale (1 = completely irrelevant, 2 = slightly relevant, 3 = moderately relevant, 4 = highly relevant), calculating item-level Content Validity Index (I-CVI) and average scale-level Content Validity Index (S-CVI/Ave) to evaluate content validity. Five items yielded an I-CVI of 1.000, and the other two scored 0.800; all values exceeded the critical cutoff of 0.780. The full scale’s S-CVI/Ave was 0.943, which passed the standard 0.900 threshold. Combined, these results confirm the items adequately measure the online class-related hope construct and reflect strong content validity.

Cronbach’s alpha was calculated to examine internal consistency; the original AEQ hope subscale reported *α* = 0.810 (Pekrun et al., 2002), whereas the revised 7-item Online Class-Related Hope Scale generated Cronbach’s *α* = 0.904 based on our formal sample of 1,294 participants, a value far above the widely accepted 0.700 cutoff and signaling excellent internal consistency. To avoid overfitting that would arise from running both exploratory factor analysis (EFA) and confirmatory factor analysis (CFA) on the same dataset, we separated our samples, utilizing an independent pilot sample (*N* = 326) for EFA and preliminary item screening and reserving the full formal sample (*N* = 1,294) exclusively for CFA. We first administered the Kaiser-Meyer-Olkin (KMO) test and Bartlett’s test of sphericity to check whether the data met factor analysis prerequisites, with results showing KMO = 0.917 and Bartlett’s approximate *χ*^2^ = 4953.167 (*df* = 21, *p* < 0.001), which indicated strong inter-item correlations and confirmed the dataset’s suitability for factor extraction. We extracted factors using principal component analysis with oblique rotation, identifying a clear unidimensional factor structure with an eigenvalue of 4.529 that explained 64.699% of total cumulative variance; all seven items produced factor loadings above 0.700, reflecting a stable, robust single-factor structure.

We next performed CFA on the unidimensional 7-item Online Class-Related Hope Scale with the full sample (*N* = 1,294) to assess model fit and convergent validity, specifying a baseline single-factor measurement model without correlated error paths. The core fit statistics were *χ*^2^ = 178.982, *df* = 14, *p* < 0.001, *χ*^2^/*df* = 12.784, GFI = 0.960, AGFI = 0.919, CFI = 0.967, TLI = 0.950, IFI = 0.967, NFI = 0.964, RMR = 0.021, SRMR = 0.031, RMSEA = 0.095, 90% CI [0.083, 0.108], PGFI = 0.480, PNFI = 0.643, PCFI = 0.644. All incremental fit indices including CFI, TLI, NFI, and IFI exceeded the standard 0.900 cutoff, and both GFI and AGFI also met this benchmark; SRMR and RMR fell below the 0.080 threshold, pointing to acceptable overall model fit. Although the RMSEA point estimate (0.095) slightly exceeds the conventional 0.080 threshold, short unidimensional scales with low degrees of freedom (*df* = 14 in the present study) tend to produce upward-biased RMSEA values owing to semantically overlapping items. The lower bound of the 90% confidence interval (0.083) also marginally surpasses this cutoff; nevertheless, complementary fit indices (CFI = 0.967, TLI = 0.950, SRMR = 0.031) satisfy ideal benchmarks. Taken together, evidence across multiple fit indices suggests the model is sufficiently acceptable for hypothesis-testing purposes. The elevated *χ*^2^/*df* ratio and borderline RMSEA value stemmed mainly from high semantic overlap among items measuring a single pre-class online emotional construct, alongside the well-documented sensitivity of chi-square statistics to large sample sizes, yet comprehensive evaluation of all core fit indices and convergent validity outcomes confirmed the unidimensional measurement structure was acceptable for subsequent analytical steps.

Convergent validity tests showed standardized item loadings ranged from 0.677 to 0.813, all exceeding the 0.500 cutoff with every path coefficient statistically significant at *p* < 0.001, and the latent variable yielded Average Variance Extracted (AVE) = 0.576 and Composite Reliability (CR) = 0.905, satisfying the standard criteria of AVE > 0.500 and CR > 0.700 and verifying strong convergent validity. Finally, we applied the Fornell-Larcker criterion to test discriminant validity across the three latent constructs of online class-related hope, external motivation, and learning engagement, finding the square root of the AVE for online class-related hope reached 0.759, a value higher than all bivariate correlation coefficients between this latent variable and the other two constructs, which confirmed adequate discriminant validity of the scale within our sample.

#### 2.3.2 External motivation scale

External motivation was measured via the external factors subscale from the Inventory of School Motivation (ISM), which McInerney et al. (2003) and McInerney and Ali (2006) developed based on goal theory. The original scale encompasses eight first-order factors including task, effort, competition, social influence, affiliation, social concern, praise, and token rewards alongside four second-order higher-order dimensions of mastery, social, performance, and external factors, so this study selected the praise and token rewards first-order subscales to operationalize the overarching external motivation construct capturing students’ drive to pursue learning for external recognition and tangible rewards. The instrument uses a 5-point Likert response format (1 = strongly disagree, 5 = strongly agree), with representative items including “Receiving praise from classmates for strong academic performance matters a lot to me” from the praise dimension and “Praise alone is not enough for good grades; I value tangible rewards” from the token reward dimension; its Chinese translation and revision strictly followed the standardized translation—back-translation–pilot testing workflow, where two psychology researchers independently translated the original English items and synthesized a unified initial draft before a cross-cultural psychology scholar completed back-translation and semantic comparison to eliminate meaningful interpretive gaps. Thirty college students with online learning experience then took part in a pilot test, and item wording was refined based on their feedback to guarantee readability and alignment with online learning scenarios.

Reliability analyses demonstrated robust internal consistency: the original ISM recorded Cronbach’s *α* values of 0.770 for the praise subscale and 0.720 for the token reward subscale (McInerney and Ali, 2006), while our sample of 1,294 valid responses yielded *α* = 0.870 for praise and *α* = 0.830 for token rewards, both of which surpassed the conventional 0.700 cutoff and fully met psychometric criteria. We subsequently conducted confirmatory factor analysis to examine the construct structure of external motivation by first estimating a two-factor model treating praise and token rewards as separate latent variables, yet this initial model exhibited severe fit deficiencies and failed all discriminant validity benchmarks. The standardized correlation between the two latent constructs reached 0.850, a figure higher than the square root of each dimension’s AVE (0.759 for praise, 0.660 for token rewards), and the HTMT ratio hit 1.000 alongside an MSV value exceeding both AVE estimates, which collectively signified a complete violation of discriminant validity rules. Compounding these issues, global model fit was extremely poor, with *χ*^2^/*df* = 36.685, RMSEA = 0.166, CFI = 0.804, and TLI = 0.756; all core fit statistics fell below acceptable thresholds, revealing extreme multicollinearity that indicated the two dimensions reflected a single unified latent construct rather than distinct separate factors.

To resolve these structural flaws, we refined the measurement model through a combined theory-driven and data-driven approach, starting with integrating praise and token rewards into one single latent variable labeled external motivation, a step that fundamentally fixed the discriminant validity failure stemming from their high empirical and theoretical overlap. We then carried out sequential item purification to remove indicators with weak loadings and poor theoretical fit: item token 9 was eliminated for its low standardized loading of 0.364 and limited explanatory power; we further dropped token 4 and token 40, whose loadings fell below 0.600 and SMC values were less than 0.300. This screening retained nine high-quality items with standardized factor loadings no lower than 0.696, satisfying basic convergent validity requirements, after which we further improved model fit by adding theoretically defensible error co-variances guided by modification indices. We specified a covariance path between praise 10 and praise 13, two items both measuring participants’ valuation of academic praise from different sources that share homologous content and naturally carry correlated residual variance, and a second covariance path between token 1 and token 41, whose matching framing around reward-driven learning effort similarly justified correlated residuals.

Fit statistics for the optimized single-factor measurement model were *χ*^2^ = 602.008, *df* = 25, *χ*^2^/*df* = 24.080, GFI = 0.906, RMSEA = 0.134, SRMR = 0.048, CFI = 0.925, TLI = 0.891, IFI = 0.925. CFI, GFI, IFI, and SRMR all satisfied the ideal fit standards proposed by Hu and Bentler (1999), and TLI of 0.891 closely approached the widely adopted 0.900 cutoff. For this nine-item single-factor model (*df* = 25), the moderately high RMSEA (0.134) is likely inflated by the large sample size. Nevertheless, all incremental fit indices (CFI = 0.925, IFI = 0.925, GFI = 0.906) meet conventional benchmarks, and SRMR = 0.048 falls well below the 0.080 cutoff, jointly confirming acceptable model adequacy. The inflated *χ*^2^/*df* and marginal RMSEA values largely stemmed from the large sample size of 1,294, given the well-documented sensitivity of chi-square tests to big datasets, alongside repetitive sentence structures across scale items that created partial measurement overlap and elevated residual error. When evaluating all core fit indices holistically, the single-factor structure met the relaxed fit thresholds commonly accepted in social science research, confirming adequate correspondence between the measurement model and sample data. Follow-up convergent validity tests produced favorable outcomes: standardized factor loadings for all retained items ranged from 0.696 to 0.813, every loading exceeded the 0.500 cutoff, and all path coefficients carried statistically significant z-values at *p* < 0.001. The latent construct generated composite reliability (CR) = 0.910 and average variance extracted (AVE) = 0.530, satisfying the standard benchmarks of CR > 0.700 and AVE > 0.500 and confirming strong convergent validity for the external motivation scale developed in this study.

#### 2.3.3 Learning engagement scale

Drawing on the three-dimensional student engagement theory proposed by Fredricks et al. (2004), this study conceptualized learning engagement as a multidimensional latent construct covering behavioral, emotional, and cognitive engagement, and adopted the Chinese version of the School Engagement Scale originally developed by Fredricks et al. (2004) and locally revised by domestic scholars for measurement. The original scale contained 19 self-report items scored on a 5-point Likert format (1 = strongly disagree, 5 = strongly agree) including several reverse-scored items; we recoded all reversed items using the formula 6 − raw score prior to formal analysis to unify scoring direction, and all subsequent statistical analyses relied entirely on this adjusted dataset. We then performed stepwise confirmatory factor analysis on the full sample to optimize model fit and eliminate low-contribution observed indicators, removing items with low standardized loadings, excessive cross-loadings and abnormal residuals and retaining 13 valid items consisting of four behavioral engagement items, five emotional engagement items and four cognitive engagement items to form the three-factor measurement model, with representative statements including “I follow the rules at school” for behavioral engagement, “I enjoy going to school” for emotional engagement, and “If I do not understand what I read, I read it again” for cognitive engagement.

Confirmatory factor analysis based on the valid sample of *N* = 1,294 was conducted on the refined 13-item three-factor structure, and all incremental fit indices including CFI = 0.928, TLI = 0.910, NFI = 0.924 and IFI = 0.928 as well as SRMR = 0.040 met widely accepted standards, while *χ*^2^/*df* = 17.164 and RMSEA = 0.112 (90% CI = [0.106, 0.118]) failed strict cutoff criteria. Consistent with modern SEM recommendations, we prioritize incremental fit indices and SRMR over RMSEA for multi-factor educational scales. CFI = 0.928, TLI = 0.910, and SRMR = 0.040 all satisfy rigorous thresholds. The elevated RMSEA (0.112)—likely attributable to strong theoretical intercorrelations among the three engagement dimensions and a large sample size—does not undermine overall model fit. Since chi-square statistics are highly sensitive to sample size, the large sample of 1,294 inevitably inflated these two indicators; following the fit evaluation framework of Hu and Bentler (1999), we took incremental fit indices and SRMR as core judgment bases and deemed the three-factor structure acceptable for subsequent structural equation modeling. Convergent validity tests further supported the measurement structure, as standardized loadings of all 13 items ranged from 0.708 to 0.856 and all surpassed the 0.500 threshold with strong explanatory power for corresponding latent variables; composite reliability values for behavioral, emotional and cognitive engagement were 0.858, 0.920 and 0.883, and AVE values reached 0.603, 0.696 and 0.655 respectively, all satisfying the benchmarks of CR > 0.700 and AVE > 0.500. Reliability examination also yielded favorable results, with Cronbach’s *α* of the full scale reaching 0.960 and subscale *α* coefficients falling between 0.860 and 0.920, which met standard psychometric requirements.

We further tested discriminant validity jointly via the Fornell-Larcker criterion and HTMT method, yet several inter-construct correlations exceeded the square root of corresponding AVE values under the Fornell-Larcker standard, and HTMT ratios among the three dimensions ranged from 0.911 to 0.967, all above the strict 0.850 cutoff and even exceeding the lenient 0.900 threshold for the links between behavioral engagement and the other two constructs. Such high correlations align with the core connotation of three-dimensional engagement theory, given that behavioral, emotional and cognitive engagement are interdependent and mutually reinforcing inherent dimensions of student engagement; together with the scale’s satisfactory convergent validity and mature theoretical foundation for its three-factor division, the measurement model was judged sufficiently interpretable to support follow-up path analysis, and the overall 13-item scale demonstrated reliable internal consistency and convergent validity that matched the sample data well for measuring student learning engagement.

We also took both pre-survey procedural controls and post-hoc statistical tests to mitigate common method bias brought by single-point self-report data. During questionnaire distribution, all surveys remained anonymous to reduce social desirability bias, items from different scales were randomly interleaved to avoid fixed response tendencies, reverse-scored items were embedded to break mechanical answering habits, and clear instructions stressed there were no right or wrong answers to guide authentic responses. Harman’s single-factor test via unrotated principal component analysis was then carried out on all original scale items, extracting five factors with eigenvalues above 1 where the first unrotated factor explained only 43.518% of total variance, demonstrating that common method bias would not seriously interfere with the study’s core conclusions when combined with the full set of procedural precautions. It should be noted that all Cronbach’s *α* and composite reliability values reported in this section were calculated from the current sample of 1,294 valid responses rather than cited from original scale literature, and all three measurement scales adopted in this research presented sound reliability and validity, providing high-quality data support for hypothesis testing and structural equation modeling and ensuring the rigor and stability of all research findings.

Descriptive statistics and zero-order correlations among the three core latent constructs are summarized in Table 2.

**Table 2:** Means, standard deviations, and inter-variable correlations.

Variables	*M*	*SD*	1	2	3
1. Online class-related hope	3.513	0.666	1		
2. External motivation	3.432	0.664	0.546***	1	
3. Learning engagement	3.498	0.646	0.691***	0.703***	1

*N* = 1,294. All items were rated on a 5-point Likert scale (1 = strongly disagree, 5 = strongly agree). ***p < 0.001, two-tailed.

This study integrated pre-survey procedural safeguards and post-hoc statistical assessments to alleviate common method variance originating from single-wave self-report data. During questionnaire administration, anonymous data collection was implemented to curb social desirability bias; items from distinct scales were randomly interspersed to eliminate rigid response patterns, reverse-coded items were embedded to prevent mechanical answering, and standardized neutral instructions were provided to elicit genuine participant responses. Two complementary statistical examinations, namely the unrotated Harman single-factor test and the full collinearity variance inflation factor (FCVIF) test developed by Kock and Lynn (2012), were deployed for bias diagnosis. For the Harman single-factor test, unrotated principal component extraction generated five factors with eigenvalues exceeding 1, and the first unrotated factor‘s explained variance fell below the conventional 50% threshold. The FCVIF test served as supplementary validation. Composite mean scores were calculated for every latent construct, followed by sequential regression analyses where each construct acted as the criterion variable regressed on all remaining latent variables and the gender moderator to derive the coefficient of determination (*R*^2^) for each regression model. FCVIF values were computed via the formula *FCVIF = 1/(1 − R^2^)*, and corresponding FCVIF statistics for all latent constructs are summarized in Table 3, with all FCVIF estimates falling below the critical cutoff of 3.300. Combined evidence from procedural design, Harman’s test, and FCVIF diagnostics confirms the absence of severe common method bias within the dataset, which guarantees robust and trustworthy outputs for subsequent analytical procedures.

**Table 3:** R-squared and full collinearity variance inflation factor (FCVIF) results of latent constructs.

Latent construct	*R^2^*	FCVIF
Online class-related hope	0.486	1.946
External motivation	0.503	2.012
Learning engagement	0.630	2.703

### 2.4 Data analysis strategy

All statistical analyses in this research were conducted using IBM SPSS 27.0 and Hayes’ PROCESS Macro Version 4.1. As a cross-sectional correlational study, all research hypotheses were tested through conditional process models established based on ordinary least squares regression, following a standardized stepwise analytical procedure.

Prior to formal hypothesis testing, we carried out comprehensive preliminary checks to verify all regression assumptions. Univariate normality of observed learning engagement scores was examined via normal Q-Q plots, matrix scatterplots were generated to assess linear relationships across the three core latent constructs, and variance inflation factors VIF were calculated to detect multicollinearity. We adopted VIF < 3 as the threshold indicating no severe multicollinearity, which guaranteed stable and credible regression outputs.

We first computed composite scores for each latent construct, then calculated descriptive statistics and two-tailed bivariate Pearson correlations for the core variables to lay a foundation for regression modeling.

For simple mediation testing, online class-related hope was treated as the independent variable, external motivation served as the mediator, and learning engagement functioned as the dependent variable, with gender, academic year, and major entered as covariates. We set 5,000 bias-corrected bootstrap resamples to construct 95% CIs. A significant mediating effect was identified if the CI for the indirect effect did not contain zero.

Finally, moderated mediation models were estimated to test gender as the moderator acting on the second segment of the path (external motivation → learning engagement), where only academic year and major were retained as control variables. Conditional indirect effects and simple slopes for male and female subgroups were calculated at ±1 standard deviation of external motivation, and plotting syntax was exported to create visual figures that intuitively illustrated gender differences in the moderating effect.

## 3 Results

### 3.1 Preliminary data assumption tests

Preliminary data screening and assumption verification were conducted before hypothesis testing. As reported in Section 2.3, integrated procedural controls and two statistical tests confirmed no severe common method bias in the dataset. Next, we assessed normality, linearity, and multicollinearity for regression assumptions. All VIF values were lower than 3, indicating negligible multicollinearity among variables. Descriptive statistics and zero-order correlations for core constructs are displayed in Table 2.

**Figure 2:**
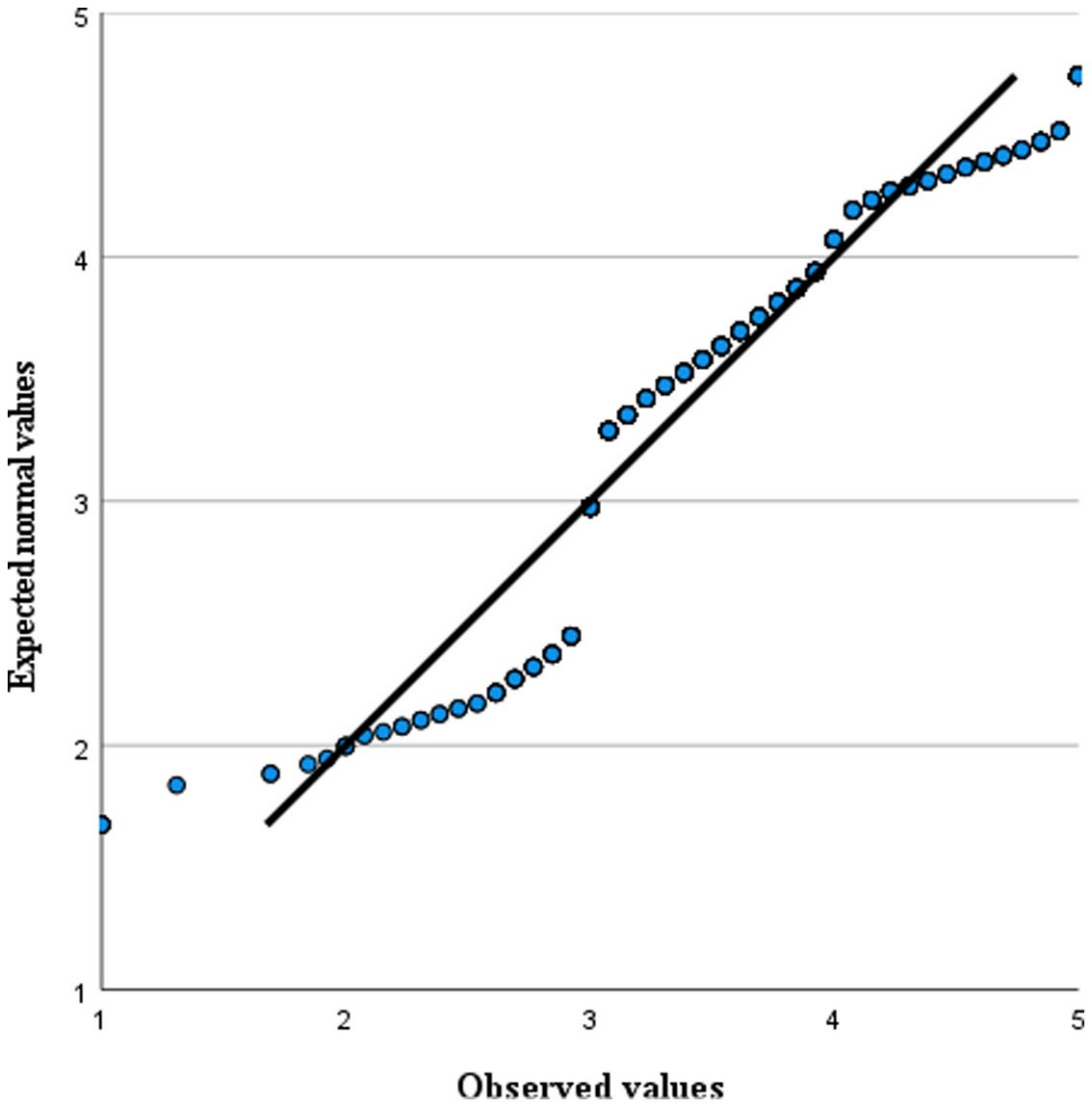
Normal Q-Q plot of learning engagement.

**Figure 3:**
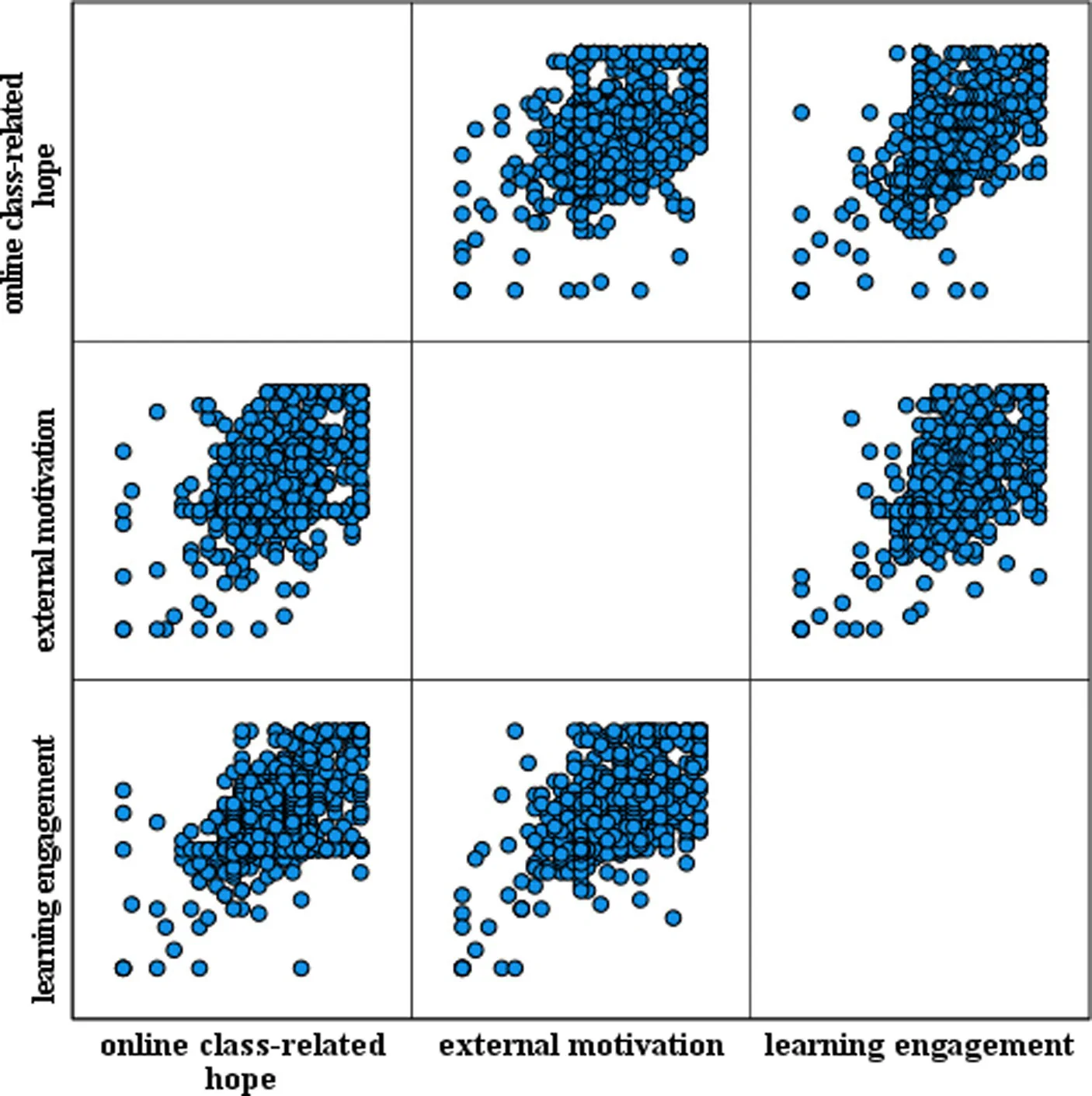
Matrix scatterplot of online class-related hope, external motivation, and learning engagement.

Prior to regression modeling, we performed a series of preliminary assumption checks for ordinary least squares regression. The normal Q-Q plot of learning engagement (Figure 2) showed nearly all data points clustered along the theoretical diagonal, with only minor deviations at the extreme tails, indicating the observed variable approximately followed a univariate normal distribution. Matrix scatterplots (Figure 3) further examined pairwise associations among online class-related hope, external motivation, and learning engagement; all pairs displayed clear positive linear trends without obvious nonlinear patterns, satisfying the linearity assumption of regression analysis. Variance inflation factors (VIF) were calculated to detect multicollinearity. As shown in Table 4, VIF values for all predictors in the two mediation regression equations ranged from 1.011 to 1.447, far below the threshold of 3, confirming the absence of severe multicollinearity and stable regression parameter estimates.

**Table 4:** Variance inflation factor (VIF) values for multicollinearity diagnosis.

Predictor variable	Model 1 (DV: external motivation)	Model 2 (DV: learning engagement)
Online class-related hope	1.011	1.447
External motivation	—	1.431
Gender	1.011	1.014
Major	1.032	1.032
Grade	1.049	1.051

Table 5 presents descriptive statistics and two-tailed Pearson bivariate correlations for all focal variables (*N* = 1,294). Significant positive correlations emerged across every pair of constructs: online class-related hope was positively correlated with learning engagement (*r* = 0.691), (*p* < 0.001) and external motivation (*r* = 0.546), (*p* < 0.001), while external motivation also exhibited a strong positive association with learning engagement (*r* = 0.703), (*p* < 0.001).

**Table 5:** Pearson correlation matrix (*N* = 1,294).

Variable	Online class-related hope	External motivation	Learning engagement
Online class-related hope	1	0.546***	0.691***
External motivation	0.546***	1	0.703***
Learning engagement	0.691***	0.703***	1

***p < 0.001 (two-tailed). All correlations are statistically significant.

### 3.2 Mediation effect test of external motivation

PROCESS Model 4 was adopted in this section to examine the mediating role of external motivation, with gender, major, and academic year entered as covariates in all regression equations.

The mediation test was implemented in three sequential steps. First, a total effect model was constructed to test the predictive effect of online class-related hope on learning engagement. The results revealed that online class-related hope significantly and positively predicted learning engagement (*B* = 0.667, *p* < 0.001). The overall regression model was statistically significant, *F*(4, 1,289) = 297.700, *p* < 0.001, *R*^2^ = 0.480, providing preliminary support for Hypothesis 1. Second, we estimated the predictive path from the independent variable to the mediator. Online class-related hope significantly positively predicted external motivation (*B* = 0.550, *p* < 0.001), and the model demonstrated satisfactory fit, *F*(4, 1,289) = 138.900, *p* < 0.001, *R*^2^ = 0.301, which validated Hypothesis 2a. Third, both online class-related hope and external motivation were simultaneously included in the regression equation to predict learning engagement. External motivation retained a significant positive predictive effect (*B* = 0.457, *p* < 0.001), and the full mediation regression model was significant overall, *F*(5, 1,288) = 445.940, *p* < 0.001, *R*^2^ = 0.634, offering preliminary evidence for Hypothesis 2b. Detailed results are displayed in Table 6.

**Table 6:** Summary of hierarchical regression results for the mediation model (*N* = 1,294).

Outcome variable(s)	Predictor variable(s)	*B*	*F*	*R* ^2^	*p*
Learning engagement	Online class-related hope	0.667	297.700	0.480	*p* < 0.001
External motivation	Online class-related hope	0.550	138.900	0.301	*p* < 0.001
Learning engagement	Online class-related hope External motivation	0.416 0.457	445.940	0.634	*p* < 0.001

Covariates gender, major, and academic year were controlled for in all regression models. *B* = unstandardized regression coefficient. All regression coefficients reported in the table were statistically significant at *p* < 0.001.

**Figure 4:**
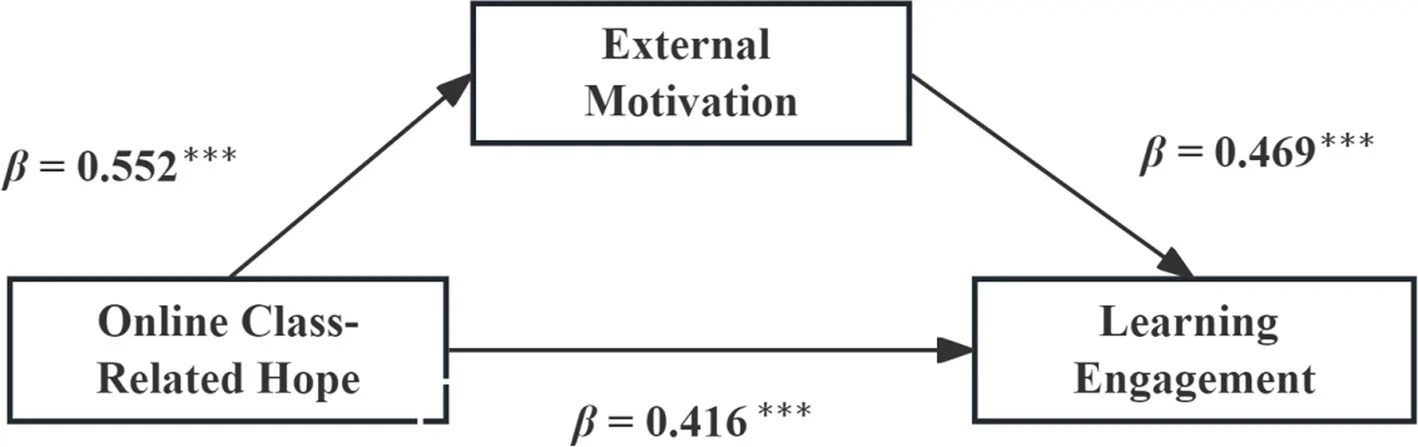
Mediation model of external motivation between online class-related hope and learning engagement. ***p < 0.001. Direct effect of online class-related hope on learning engagement = 0.416; standardized indirect effect = 0.259, Bootstrap 95% CI [0.219, 0.299].

Effect decomposition based on 5,000 bias-corrected bootstrap resamples yielded the following results. The total effect of online class-related hope on learning engagement was *B* = 0.667 (Boot SE = 0.020, 95% CI [0.629, 0.706]). The direct effect of online class-related hope on learning engagement was *B* = 0.416 (Boot SE = 0.020, 95% CI [0.378, 0.455]), accounting for 62.36% of the total effect. The indirect effect transmitted through external motivation was *B* = 0.251 (Boot SE = 0.021, 95% CI [0.211, 0.297]), representing 37.64% of the total effect. The 95% confidence interval for the indirect effect excluded zero, and the direct effect remained statistically significant. These findings confirmed that external motivation partially mediated the association between online class-related hope and learning engagement, supporting Hypothesis 2. Full detailed results are presented in Table 7 and Figure 4. Notably, given the cross-sectional design of this study, we can only identify correlational patterns among variables rather than clarify causal temporal ordering or establish rigorous causal mechanisms. Interpretations of all reported effects should therefore be approached with caution.

**Table 7:** Decomposition of total, direct, and indirect effects.

Effect type	*B*	Boot SE	95% CI	*β*
Total effect	0.667	0.020	[0.629, 0.706]	0.687
Direct effect	0.416	0.020	[0.378, 0.455]	0.429
Indirect effect	0.251	0.021	[0.211, 0.297]	0.259

*B* = unstandardized effect; *β* = completely standardized effect; Boot SE = Bootstrap standard error; CI = confidence interval (5,000 bias-corrected bootstrap samples).

### 3.3 Moderated mediation effect test

Hayes’ PROCESS Model 14 was used to estimate the moderated mediation model. Online class-related hope was specified as the independent variable, external motivation as the mediator, and gender as the moderator. Only major and academic year were included as covariates, and 5,000 bias-corrected bootstrap resamples were generated for significance testing. The overall regression model was statistically significant, *F*(6, 1,287) = 371.970, *p* < 0.001, *R*^2^ = 0.634.

Regression outputs revealed that the interaction term of external motivation and gender exerted a nonsignificant predictive effect on learning engagement *B* = 0.040, *p* = 0.238. The moderated mediation index was further calculated as Index = 0.022, with a 95% bootstrap confidence interval of [−0.025, 0.068], which contained zero. Collectively, these results indicated that gender did not significantly moderate the second half of the indirect pathway (external motivation → learning engagement), failing to support Hypothesis 3. Detailed results are presented in Table 8.

**Table 8:** Moderated mediation model results.

Predictor and index	*B*	*p*	95% Bootstrap CI
External motivation × gender interaction	0.040	0.238	[−0.027, 0.107]
Moderated mediation index	0.022	—	[−0.025, 0.068]

Covariates included major and academic year; 5,000 bias-corrected bootstrap resamples. All values rounded to 3 decimal places.

Conditional indirect effects were calculated separately for male and female subgroups. For male students, the conditional indirect effect was *B* = 0.242, 95% CI [0.201, 0.283]; for female students, the conditional indirect effect was (*B* = 0.264), 95% CI [0.209, 0.320]. Neither confidence interval spanned zero, meaning the mediating effect of external motivation remained statistically significant for both genders. However, the confidence interval of the moderated mediation index capturing the gender difference in indirect effects included zero, demonstrating no statistically meaningful disparity in the magnitude of the indirect effect between male and female participants. Detailed results are presented in Table 9.

**Table 9:** Conditional indirect effects by gender subgroup.

Group	*B*	95% Bootstrap CI
Male students	0.242	[0.201, 0.283]
Female students	0.264	[0.209, 0.320]

The confidence interval of the moderated mediation index contains zero, which means gender does not significantly moderate the indirect pathway; Hypothesis 3 was not supported. The indirect effect via external motivation is significant for both genders, with no meaningful difference in effect size between groups.

**Figure 5:**
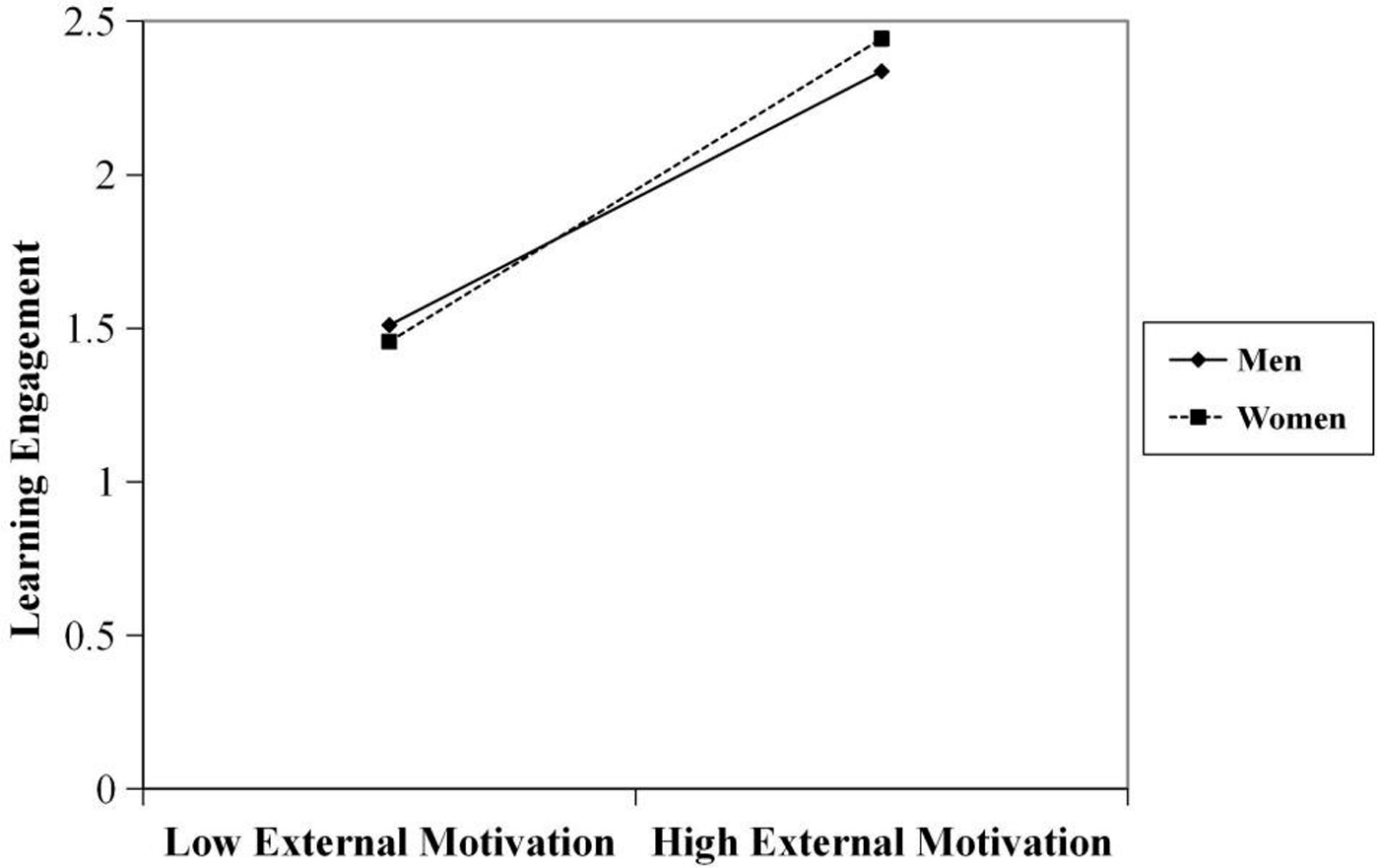
Simple slopes for external motivation predicting learning engagement by gender. Overlapping slopes (solid/diamond = male; dashed/square = female) indicate non-significant gender differences.

**Figure 6:**
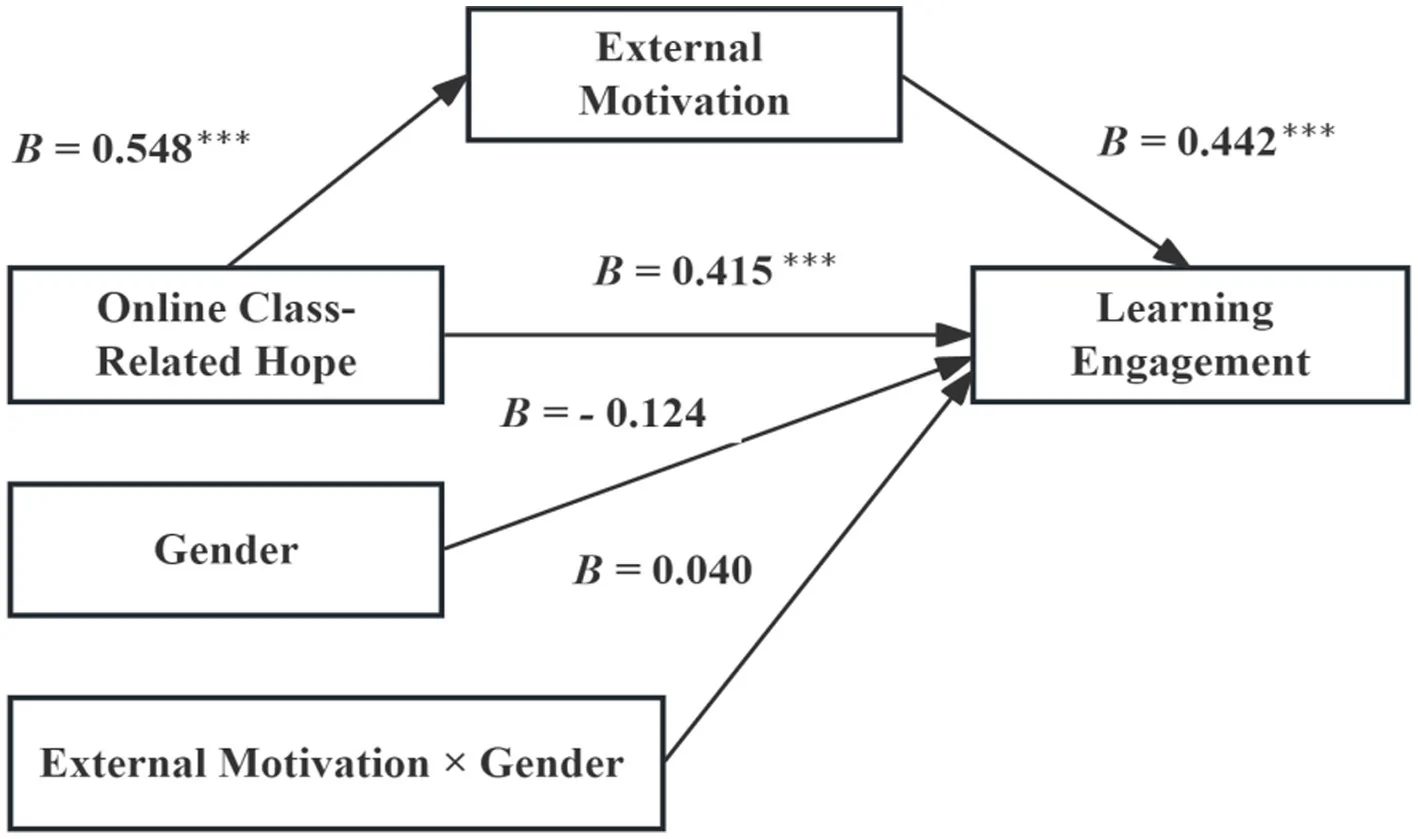
Moderated mediation model (Hayes’ Model 14; gender moderates the path from external motivation to learning engagement). Unstandardized coefficients (*B*) are shown; ***p < 0.001. Interaction: *B* = 0.040, ns. Index = 0.022, 95% bootstrap CI [−0.025, 0.068], indicating non-significant moderated mediation.

A simple slope plot (Figure 5) was created to visually illustrate these associations. The solid line represents male students, and the dashed line represents female students. The two lines exhibited nearly identical slopes with minimal separation, further corroborating the regression finding that the positive predictive strength of external motivation for learning engagement did not differ markedly by gender. Figure 6 presents the path coefficient diagram of the moderated mediation model.

## 4 Discussion

This study integrated control-value theory and self-determination theory to construct a conditional process model with external motivation as the mediator and gender as the moderator, which aimed to unpack the internal mechanism linking online class-related hope to learning engagement among college students. Given the cross-sectional self-report survey design adopted in this research, all analytical results merely reflect concurrent correlations across variables and cannot be interpreted as causal evidence.

### 4.1 Positive correlation between online class-related hope and learning engagement

Consistent with the prediction proposed in Hypothesis 1, online class-related hope exhibited a strong, significant positive correlation with learning engagement (*r* = 0.691, *p* < 0.001), and this association remained robust after controlling for the covariates of gender, academic year, and major. This finding aligns with the core logic of control-value theory: learners who perceive high controllability and value regarding academic tasks experience positive academic emotions, which in turn drive multifaceted behavioral, emotional, and cognitive learning engagement (Pekrun, 2006). Within online learning contexts specifically, students who hold hope for online courses—meaning they believe they can master course content and recognize the long-term developmental value of online learning—demonstrate greater initiative in classroom participation, stronger emotional identification with coursework, and deeper levels of cognitive processing.

Prior scholarship has documented positive links between general hope and academic outcomes such as academic performance and learning engagement (Ge et al., 2025; Tomás et al., 2020; Marques et al., 2017). However, most existing work operationalizes hope as a broad dispositional trait rather than a context-specific academic emotion unique to online instruction. The present study addresses this research gap by empirically verifying the concurrent positive association between the two constructs within digital learning environments. To date, few studies have formally distinguished general trait hope from context-specific online class-related hope as an academic emotion. By subjecting the hope subscale of the Achievement Emotions Questionnaire (AEQ) to rigorous translation, back-translation, and content validity evaluation, this study adapted the instrument for online learning settings and provided a context-specific operational definition of the focal construct.

From a practical educational standpoint, higher levels of online class-related hope correspond to more favorable self-reported learning engagement. Nevertheless, this correlational pattern cannot establish a causal claim that fostering hope directly boosts engagement. Instead, the results imply that instructional designs targeting the two core antecedents of online class-related hope—perceived controllability and task value—are likely to yield concurrent improvements in students’ self-rated learning engagement. For instance, instructors can design incremental, attainable online learning objectives and clarify the practical and developmental value of course content to solidify the cognitive foundations of students’ hopeful emotions, thereby facilitating greater online learning engagement.

### 4.2 Partial mediating role of external motivation

Hypothesis 2 received partial empirical support: online class-related hope had a significant positive correlation with external motivation, and external motivation served as a partial mediator in the relationship between online class-related hope and learning engagement among college students. Bootstrap effect decomposition revealed that the indirect effect via external motivation accounted for 37.64% of the total association between hope and learning engagement, while the direct association between the two constructs remained statistically significant, confirming the existence of partial mediation. The integrated theoretical framework built by combining control-value theory of academic emotions and self-determination theory fills a notable gap in existing literature: prior research rarely integrates these two theories to systematically unpack the sequential “emotion–motivation–behavior” linkage within online educational contexts.

From the lens of control-value theory, students’ appraisals of task controllability and task value activate the positive academic emotion of online class-related hope. The emergence of hopeful feelings, in turn, markedly heightens students’ sensitivity to external academic rewards such as grades, instructor and peer praise, and academic honors (Pekrun, 1992, 2006). Students who hold high hope for online courses are more inclined to regard outstanding academic performance, social recognition, and academic distinctions as core criteria for evaluating learning outcomes, which subsequently strengthens their external motivation to learn. Drawing on self-determination theory, external motivation is driven by external outcomes rather than intrinsic interest in learning itself, and it acts as a critical sustaining factor for college students’ persistent online learning. This supportive function of external motivation becomes especially prominent in self-directed online learning environments, where students generally possess weaker intrinsic learning drive (Ryan and Deci, 2000).

Existing studies have separately documented the pairwise links between positive academic emotions and external motivation, as well as external motivation and sustained learning efforts (Isen and Reeve, 2005; Adnan and Anwar, 2020). Nevertheless, no prior work has adopted external motivation as a mediating variable to systematically examine the internal mechanism linking context-specific online class-related hope to online learning engagement. This study empirically verified the partial mediating role of external motivation and quantified the proportional magnitudes of direct and indirect pathways. The core findings indicate two parallel pathways underlying the association between online class-related hope and learning engagement: a direct engagement pathway rooted in positive academic emotions, and an indirect motivational pathway fueled by external rewards.

### 4.3 Non-significant gender moderation effect

Contrary to Hypothesis 3, gender failed to exert a significant moderating influence on the association between external motivation and learning engagement. The regression coefficient for the interaction term of external motivation and gender was nonsignificant (*B* = 0.040, *p* = 0.238), and the 95% confidence interval for the moderated mediation index contained zero. Simple slope plots further illustrated nearly parallel regression lines for male and female subgroups, with no meaningful gender gaps in engagement disparities across high and low levels of external motivation. These results confirmed that the strength of the link between external motivation and learning engagement did not differ significantly by gender. The indirect pathway via external motivation remained statistically significant for both male and female students, and the magnitude of this indirect effect showed no statistically meaningful divergence between the two groups.

This nonsignificant gender moderation finding adds nuanced contradictory evidence to the mixed body of existing research on gender disparities in external learning motivation. Several prior studies have argued that female students tend to respond more strongly to academic praise and tangible external rewards (Ardeńska et al., 2019; Kuśnierz et al., 2020), whereas other scholarship suggests male learners are more readily driven by competitive external incentives (Naz et al., 2020). Three key contextual and methodological distinctions explain these inconsistent cross-study outcomes and strengthen the reliability of the present results. First, the unique online learning context distinguishes this work from prior gender-motivation research, which predominantly centers on in-person classroom settings. Uniform online feedback channels and standardized grade reporting systems dilute gender-based disparities in perceptions of external rewards, narrowing gaps between male and female students’ responsiveness to external incentives. Second, supplementary robustness tests rule out confounding bias from uneven gender distribution within the full sample (368 male participants, 926 female participants). Even after conducting matched subsample robustness checks, the nonsignificant moderation result persisted, eliminating sample imbalance as a plausible alternative explanation. Third, the refined measurement instrument creates methodological divergence from earlier work. This study resolved structural flaws in the original Inventory of School Motivation by merging the separate praise and token reward subdimensions into a unified external motivation construct, which fixed the original scale’s poor discriminant validity. Most previous gender comparisons retained the two-factor subscale structure, a critical source of conflicting empirical conclusions across the literature.

Within the online student sample examined here, external incentives foster learning engagement to an equivalent degree across gender groups under remote instructional environments. This practical implication suggests educators do not need to design gender-segregated external reward or incentive frameworks for online courses, as the positive association between external motivation and online learning engagement holds uniform strength for male and female learners alike.

### 4.4 Theoretical contributions

This study delivers two major incremental theoretical contributions to the research domain of online academic emotions and learning motivation.

First, the present work integrates two classic theories to develop a context-specific conditional process model tailored for online learning environments. Prior scholarship tends to adopt either control-value theory of academic emotions or self-determination theory in isolation, resulting in fragmented theoretical applications. This study establishes an associative pathway of online class-related hope → external motivation → learning engagement and empirically verifies the synergistic interplay between control-value appraisals of academic emotions (rooted in control-value theory) and the regulatory mechanism of external motivation (derived from self-determination theory). These findings refine the theoretical analytical framework depicting the emotion–motivation–learning behavior chain within digital learning contexts.

Second, this research refines the construct measurement and nomological network of online class-related hope. Earlier investigations mostly rely on general dispositional hope scales that fail to fit the unique demands of online instruction. Through rigorous translation and back-translation procedures, expert content validity assessment, exploratory factor analysis based on an independent pilot sample, and confirmatory factor analysis using the full formal dataset, we developed a reliable and valid 7-item unidimensional Online Class-Related Hope Scale. This work clarifies the conceptual connotation and standardized measurement criteria of this context-specific academic emotion, supplying a standardized measurement instrument for future inquiries into online academic emotions.

### 4.5 Practical educational implications

As a cross-sectional self-report investigation, this study cannot confirm causal effects of instructional interventions. All practical recommendations drawn from the results are grounded solely in concurrent correlational patterns observed among the measured variables. Longitudinal tracking or experimental designs are required in future research to further validate the effectiveness of these actionable strategies. Detailed practical implications are outlined below.

Educators should prioritize nurturing students’ online class-related hope to boost overall learning engagement. Instructors may break online curricula into progressive modular tasks supplemented with step-by-step learning guidance and timely asynchronous Q&A support to strengthen students’ perceived controllability over online coursework. Meanwhile, teachers ought to articulate clear connections between course content and career advancement, professional practice, and postgraduate prospects to raise students’ perceived task value. Such practices activate hopeful academic emotions, which in turn drive higher levels of learning engagement.

Educators may also deploy external motivation al incentives strategically to build diversified online reward systems. External motivation and hopeful emotions operate synergistically to predict greater learning engagement, so instructors can integrate external reinforcements such as written praise, periodic tangible rewards, and transparent grading rubrics alongside intrinsically motivating activities including online seminars and inquiry-based assignments. This balanced design harmonizes internal and external learning drives and prevents overreliance on external rewards that might erode autonomous learning initiative.

Finally, gender-differentiated incentive structures are unnecessary within online instructional environments. Given the nonsignificant moderating effect of gender observed in this study, unified external reward frameworks work equally well for male and female learners. Instead of customizing reward mechanisms by gender, online educators can prioritize personalized pacing adjustments, collaborative group activities, and targeted emotional support to improve the overall online learning experience for all students.

### 4.6 Limitations and future research directions

This study contains several methodological and contextual limitations that constrain causal inference and the generalizability of its findings, which can be addressed in follow-up research through targeted improvements.

First, the cross-sectional single-wave design limits causal conclusions. All constructs were measured at one identical time point, making it impossible to establish temporal ordering or directional causal relationships between online class-related hope, external motivation, and learning engagement. Reciprocal and cyclical bidirectional associations likely exist across these three variables. Future scholars may adopt longitudinal panel designs or randomized controlled intervention trials to rigorously identify directional predictive pathways and underlying mechanistic processes.

Second, exclusive reliance on cross-sectional self-reported survey data introduces potential social desirability bias and inherent common method variance concerns. Though we mitigated common method bias via procedural safeguards including anonymous administration, randomized item ordering, and embedded reverse-coded items, alongside post-hoc statistical checks: Harman’s single-factor test showed the largest unrotated factor explained less than 50% of total variance, and full collinearity diagnostics indicated all FCVIF values fell below the critical cutoff of 3.300. These conventional remedies are relatively conservative and cannot fully eliminate single-source measurement error. We acknowledge that Harman’s test and FCVIF analysis merely serve as preliminary screening tools with limited sensitivity to systematic method bias. For more robust control of common method variance, future research may adopt rigorous designs such as marker variable approaches or multi-wave time-lagged surveys. Additional improvements can incorporate objective digital learning metrics—including online learning duration, assignment completion rates, and forum interaction frequency—to realize data triangulation between subjective self-perceptions and objective behavioral records, further alleviating single-measurement bias.

Third, the measurement models of several core constructs produced RMSEA values higher than the conventional 0.080 benchmark. Methodological research has repeatedly noted that RMSEA tends to be artificially inflated under conditions consistent with our study: low degrees of freedom, semantically overlapping items in short unidimensional scales, strong theoretical correlations between multi-dimensional latent factors, and large samples that magnify residual-based discrepancy statistics. Rather than relying on RMSEA as a standalone criterion, we followed contemporary SEM guidelines to evaluate model fit via a holistic set of complementary indices. Across all three measurement models, incremental fit indices (CFI, TLI, IFI, and GFI) and the absolute residual index SRMR consistently met or exceeded standard thresholds for social science research. This consistent convergence across multiple fit metrics provides robust empirical evidence that the scales possess adequate psychometric properties for structural parameter estimation and hypothesis testing. Detailed fit statistics and scale-specific diagnostics are fully presented in the scale psychometrics subsection. To avoid redundant repetition, we omit itemized numerical breakdowns here and instead highlight the overall consistent pattern validating our subsequent analytical steps. Fifth, the motivational scope of the current analytical framework is relatively narrow. This study solely focuses on external motivation as the mediating variable and does not incorporate intrinsic motivation or a motivation outlined in Self-Determination Theory. Follow-up research can integrate multiple motivational subtypes to compare their distinct mediating magnitudes and construct a more holistic emotion-motivation-behavior theoretical framework for online learning contexts.

Sixth, the adapted Online Class-Related Hope Scale measures generalized trait-like hopeful tendencies toward coursework instead of momentary dynamic emotional fluctuations. The 7-item unidimensional scale cannot capture real-time state hope shifts across separate online sessions or different learning phases. Future studies may combine real-time affective monitoring and short daily micro-survey tracking to capture situational hope dynamics and unpack their correlational links with momentary learning engagement.

Despite these acknowledged limitations, the current work yields two distinct, valuable advances for the field of online educational psychology. Theoretically, it bridges Control-Value Theory and Self-Determination Theory to build a complete moderated mediation pathway that illustrates how trait-oriented online class-related hope shapes learning engagement via external motivation, addressing the fragmented separate application of these two classic frameworks in prior digital learning research. In addition, this study develops and fully psychometrically validates a specialized unidimensional Online Class-Related Hope Scale, filling the measurement gap for context-specific achievement hope within online education scholarship. Practically, the results deliver concise, gender-neutral instructional tactics centered on boosting students’ perceived control and task value of online courses to sustain learning engagement, providing actionable design guidance for college online teaching. Overall, this research expands the theoretical boundary of online achievement emotion research and offers targeted practical recommendations for postsecondary online instruction.

## 5 Conclusion

Drawing on control-value theory of academic emotions and self-determination theory, this cross-sectional questionnaire study recruited 1,294 Chinese undergraduate students to systematically test a moderated mediation model encompassing online class-related hope, external motivation, gender, and learning engagement. The core empirical findings are summarized as follows.

First, online class-related hope exhibited a significant positive concurrent correlation with online learning engagement, supporting Hypothesis 1. Students who reported higher levels of online class-related hope demonstrated greater behavioral, emotional, and cognitive engagement within online learning environments.

Second, external motivation functioned as a partial mediator in the association between online class-related hope and learning engagement, which validated Hypothesis 2. Two parallel pathways underpin this positive relationship: a direct emotional pathway through which online hope positively predicts learning engagement, and an indirect incentive pathway mediated by external motivation, with the indirect effect accounting for 37.64% of the total effect.

Third, gender failed to significantly moderate the pathway linking external motivation to learning engagement, leading to the rejection of Hypothesis 3. The strength of the positive association between external motivation and online learning engagement did not differ meaningfully between male and female participants, and the magnitude of the indirect mediating effect was nearly identical across gender subgroups.

The present study delivers two key theoretical contributions. By integrating two foundational psychological theories, it establishes an integrated emotion–motivation–behavior pathway model tailored to undergraduate online education contexts. Additionally, the standardized Online Classroom Hope Scale developed in this work enriches the theoretical landscape of digital learning emotions and motivational research. Practically, the findings offer cautious, actionable references for improving online instruction at universities. Educators may foster students’ positive online hopeful emotions by refining instructional design to enhance perceived controllability and task value; they can also deploy diversified external incentive systems to boost learning engagement. Meanwhile, gender-specific external reward frameworks are unnecessary, and instructional adjustments should prioritize universal, personalized online learning strategies instead.

Several limitations constrain rigorous causal inference and the broad generalizability of these results, including the cross-sectional research design, exclusive reliance on self-report surveys, and non-probability convenience sampling. Future research may adopt longitudinal panel tracking, experimental intervention designs, and multi-source data triangulation to further unpack causal mechanisms among focal variables, expand sample diversity, and explore additional motivational and contextual boundary conditions that shape college students’ online learning engagement.

## Data Availability

The original contributions presented in the study are included in the article/supplementary material, further inquiries can be directed to the corresponding author.
